# Beyond TG‑43: A PRISMA‐based systematic review on model‐based dose‐calculation algorithms in brachytherapy

**DOI:** 10.1002/acm2.70627

**Published:** 2026-05-20

**Authors:** Dat Tran, Thanh‐Tai Duong, Shada Wadi‐Ramahi, David Bradley, James C. L. Chow

**Affiliations:** ^1^ Department of Physics University of Houston Houston Texas USA; ^2^ Department of Medical Physics Faculty of Medicine Nguyen Tat Thanh University Ho Chi Minh City Vietnam; ^3^ Department of Radiation Oncology UPMC Hillman Cancer Center University of Pittsburgh Pittsburgh Pennsylvania USA; ^4^ Centre for Applied Physics and Radiation Technologies Sunway University Bandar Sunway Malaysia; ^5^ School of Mathematics and Physics University of Surrey Guildford UK; ^6^ Department of Radiation Oncology University of Toronto Toronto Canada; ^7^ Radiation Medicine Program Princess Margaret Cancer Centre University Health Network Toronto Canada

**Keywords:** AAPM TG‐43, Acuros BV, adaptive radiotherapy, artificial Intelligence, brachytherapy, clinical implementation, dose calculation accuracy, model‐based dose‐calculation algorithm, Monte Carlo simulation, PRISMA

## Abstract

**Background and purpose:**

The AAPM TG‐43 formalism has long served as the clinical standard for brachytherapy dose calculation but assumes a homogeneous water equivalent medium, overlooking limited scattering conditions and tissue heterogeneities. Model‐based dose‐calculation algorithms (MBDCAs), including Monte Carlo (MC) simulations overcome these limitations by accounting for real tissue composition, scatter, and applicator attenuation. This systematic review evaluates TG‐43, MBDCAs/MC methods in terms of dosimetric accuracy, validation strategies, computational feasibility, clinical implementation barriers, and emerging innovations.

**Methods:**

A PRISMA‐guided literature search was conducted using the Scopus database, identifying 284 records, of which 42 full‐text studies met inclusion criteria. Eligible studies compared at least two of the three dose‐calculation approaches (TG‐43, MBDCAs and MCs) in pelvic, breast, or head‐and‐neck brachytherapy. Extracted data encompassed dosimetric discrepancies, validation approaches, computational performance, workflow integration, and enabling technologies.

**Results:**

Across anatomical sites, TG‑43 showed no consistent bias. Its differences from heterogeneity‑aware models depended on tissue composition, scatter conditions, and source geometry. In soft‑tissue regions with minimal heterogeneity, TG‑43 generally overestimated target coverage by about 0.5%–5%. Near low‑density interfaces or in reduced‑scatter configurations, TG‑43 could instead yield lower doses than Monte Carlo or MBDCAs. For OARs, discrepancies were site‑specific: skin dose was often overestimated, while other organs showed smaller or opposite variations. Overall, MBDCAs and Monte Carlo agreed with experimental or benchmark data within roughly 3% and produced more reliable biological metrics. GPU‑accelerated and deep‑learning engines reduced computation times from hours to seconds, shifting remaining challenges toward standardization, commissioning, and QA. Successful clinical adoption relied on TG‑186–aligned validation, staff training, and integrated automated workflows.

**Conclusion:**

Evidence from the systematic review supports a clinical transition toward TG‐186–compliant, heterogeneity‐aware dose‐calculation frameworks. MBDCAs/MC algorithms provide superior dosimetric and radiobiological accuracy and are increasingly compatible with adaptive and biologically guided planning. Broad implementation is now supported by established QA standards and benchmarking datasets, which connects dosimetric precision with patient outcomes. The transition beyond TG‐43 marks a pivotal step toward precision, safety, and personalization in modern brachytherapy.

## 1 INTRODUCTION

Brachytherapy utilizes sealed radioactive sources placed within or adjacent to a target volume to achieve highly localized dose distributions with steep dose gradients.^1^, ^2^, ^3^ Clinically, it remains a cornerstone of definitive and adjuvant radiotherapy for cervical, prostate, breast, and head‐and‐neck cancers.^4^ Dose calculations for brachytherapy have relied on the formalism established by the American Association of Physicists in Medicine (AAPM) Task Group 43 (TG‐43).^5^, ^6^ This widely adopted protocol assumes an infinite, homogeneous water medium surrounding the source, thereby assuming full scattering conditions and charged particle equilibrium. Moreover, this assumption also neglects tissue heterogeneities such as variable tissue densities, bony structures, air cavities, tissue interfaces, and applicator attenuation.^7^, ^8^, ^9^, ^10^ Mazur et al.^11^ recalculated the plans of 100 patients previously treated with the SAVI applicator to the breast using AcurosBV (AXB) (Varian Medical Systems, USA) and found that in all cases the doses calculated to the PTV were lower than previously calculated using TG‐43. The differences in 20% of the plans were significant enough that it changed the coverage to “less than expected”. Placidi et al.^12^ used the advanced collapsed cone engine (ACE) (Elekta Stockholm, Sweden) and recalculated 10 plans of patients treated to the eyelids. They found that tumor coverage is less than expected and changed clinical decision necessitating the use of bolus. Similarly, Rossi et al.^13^ reported underdosage in the treatment of scalp by up to 23% when comparing the TG‐43 calculation to ACE or Monte Carlo (MC)calculations, requiring the use of bolus. Other authors reported similar results for different types of brachytherapy treatment sites.^14^, ^15^ While TG‐43 provides a simple and standardized dosimetry framework, its assumptions can lead to significant discrepancies between calculated and delivered doses in clinical scenarios.^13^, ^16^

To overcome the limitations of TG‐43, advanced model‐based dose calculation algorithms (MBDCAs) have been introduced. In 2012, AAPM Task Group 186 (TG‐186) formally recommended the clinical implementation of MBDCAs that can account for limited scattering conditions, tissue heterogeneities and applicator attenuation.^7^ Notable examples include deterministic grid‐based Boltzmann solvers such as Varian's AXB and Elekta's ACE, which utilize patient CT imaging to compute dose‐to‐medium with density heterogeneity corrections resulting in more accurate dose distributions than TG‐43, particularly in situations involving high‐atomic‐number materials or interfaces between tissue and air.^17^, ^18^ MC simulation has long been regarded as the gold standard for brachytherapy dose calculation due to their ability to model complex radiation transport physics with minimal approximations.^19^ According to the AAPM TG‐186 report,^7^ MC simulation is formally classified as a MBDCA, as it solves the Boltzmann transport equation through stochastic particle transport. For the purpose of our review, the MC methods are treated separately from deterministic MBDCAs (e.g., Acuros BV and ACE) to facilitate comparison across three practical categories encountered in the literature: (1) TG‐43 water‐based formalism, (2) deterministic MBDCAs implemented in treatment planning systems, and (3) MC simulations primarily used for benchmarking and validation. MC techniques have been applied in brachytherapy since the early 1980s, offering highly precise dose predictions by explicitly simulating millions of particle interactions in realistic geometries.^20^ This approach is free from many experimental uncertainties and can distinguish primary from scattered radiation components. However, MC simulations typically require extensive computation and meticulous validation against measurements, which limit their routine clinical use in many cases.^21^ Recent advances in computing hardware and software—for example, Graphics Processing Unit (GPU)‐accelerated MC codes—have dramatically reduced calculation times, increasingly enabling MC‐based dose calculations within clinically acceptable timeframes. These developments have shifted the remaining challenges from raw computation speed to issues of workflow integration, commissioning, and quality assurance (QA) for MC and other model‐based methods.^22^, ^23^, ^24^

Although several advanced dose‐calculation algorithms are now available, the TG‐43 formalism continues to serve as the primary standard for brachytherapy dose calculation. Adoption of MBDCAs and MC tools has been gradual and inconsistent, owing to implementation challenges such as the need for extensive commissioning, new clinical workflow, and new QA protocols. To date, several review articles have addressed parts of this evolving landscape, but with important limitations.^17^, ^18^, ^25^, ^26^ For instance, Enger et al.^17^ and Yousif et al.^18^ reviewed model‐based algorithms and their dosimetric differences relative to TG‐43. However, these were narrative reviews that focused on technical dose differences and offered only limited discussion of clinical workflow implications or QA considerations. More recently, Adhikari et al.^21^ conducted a PRISMA‐based review exclusively on MC applications in brachytherapy. While comprehensive in its scope of MC techniques, that review did not directly compare MC methods with TG‐43 or deterministic MBDCAs, nor did it address clinical impact, biological outcome metrics, or practical implementation barriers. To our knowledge, no prior systematic review has synthesized the evidence across all dose calculation approaches (TG‐43, MBDCAs/MC) within a unified comparative framework. Furthermore, emerging innovations—such as AI for rapid dose prediction, GPU‐powered dose calculations, and adaptive brachytherapy planning—are on the horizon but have not yet been addressed in prior reviews. Given this gap in literature, a systematic review is timely.

The purpose of this systematic literature review is to evaluate and compare the TG‐43 formalism, MBDCAs, and MC methods in brachytherapy aiming to provide an evidence‐based foundation to guide the transition beyond TG‐43 and to identify future directions for research and clinical practice. A complete list of abbreviations used throughout this manuscript is provided in Appendix A.

## 2 MATERIALS AND METHODS

### 2.1 Literature search strategy

A systematic literature search was conducted to identify studies based on the preferred reporting items for systematic reviews and meta‐analyses (PRISMA) guidelines.^27^ The search was performed on September 12, 2025, using the Scopus electronic database. The search strategy employed a structured combination of keywords organized into four main groups (see Appendix C for the full search string):

1. Brachytherapy: Including related terms covering methods and clinical applications.
2. Dose Calculation: Including TG‐43, MBDCAs, and MC.
3. Clinical, Biological, and Validation Outcomes: These keywords relate to the evaluation and verification of dosimetric algorithms, including clinical outcome metrics, radiobiological models, and QA techniques.
4. Feasibility, Adoption, and Emerging Innovations: Addresses the practical implementation, adoption challenges, and future innovations in brachytherapy, such as AI, GPU acceleration, and adaptive treatment planning.

### 2.2 Inclusion and exclusion criteria

All identified records were subjected to a two‐stage evaluation process based on predefined eligibility criteria. The first stage was abstract screening, titles and abstracts were reviewed for inclusion criteria, and the second stage is full‐text appraisal.

To reduce the risk of selection bias, two reviewers independently conducted the screening process at both stages. A study was included only when both reviewers reached agreement; disagreements were resolved through discussion, and if consensus could not be achieved, by consultation with a third reviewer. In addition, a risk of bias assessment was performed for all included studies, as detailed in 2.3.

For inclusion in this review, studies were required to satisfy the following four primary criteria:

- Purpose: The study's primary objective must be to compare, validate, or evaluate the performance, accuracy, feasibility, or clinical implementation of brachytherapy dose calculation methods.
- Method: The study compares at least two of the following: TG‐43, MBDCAs, or MC. This also includes studies that experimentally validate or benchmark MBDCAs or MC methods against established standards.
- Results: The study must report quantitative or qualitative results relevant to at least one of the research questions. This includes, but is not limited to, dosimetric comparisons (e.g., D90, D2cc), biological dose metrics (e.g., BED, EQD2), experimental validation data, computational performance metrics (e.g., calculation time), or analysis of clinical adoption factors.
- Other: The publication must be a full‐text, peer reviewed article written in English. The study's clinical context must be brachytherapy for pelvic, breast, or head‐and‐neck cancers. Conference abstracts, editorials, and non‐English articles will be excluded.

### 2.3 Risk of bias assessment

To evaluate the robustness and reliability of the selected studies, a risk of bias assessment was performed using four specific domains:

1. Geometry and case representativeness: evaluating how well the patient population or phantom reflects the full range of anatomical variability and complexity encountered in clinical settings.
2. Input modeling and material assignment: examining the accuracy of translating patient anatomy into digital model used for dose calculation.
3. Outcome measurement and validation: assessing the quality and appropriateness of the metrics used to quantify differences.
4. Selective outcome reporting: evaluating the transparency and completeness of the reporting.

This process was conducted by two independent reviewers, who assigned a risk level to each study across the four domains. Risk was categorized as low (+), moderate (++), or high (+++). Following independent assessment, the results were cross‐checked, and any discrepancies were discussed and resolved by consensus.

Each selected study was then evaluated based on its capacity to address the specified research questions. A summary of the contributions of the included studies to these questions is provided in Appendix B. The primary research questions (RQs) of this systematic literature review are as follows:

- RQ1: In patients undergoing brachytherapy for pelvic, breast, or head‐and‐neck cancers, how do TG‐43, MBDCAs, and MC methods compare in terms of dosimetric accuracy, heterogeneity corrections, and clinical translation into biological dose metrics?
- RQ2: What experimental validation strategies have been reported for assessing the accuracy and reliability of MBDCAs and MC compared with TG‐43 in brachytherapy treatment planning?
- RQ3: What evidence exists on the computational requirements, processing times, and workflow implications of implementing MBDCAs and MC methods in clinical practice, and how do these compare with the established TG‐43 formalism?
- RQ4: What barriers and enablers influence the clinical adoption of MBDCAs and MC methods in brachytherapy?
- RQ5: What emerging innovations have been reported to enhance accuracy, efficiency, or personalization in brachytherapy dose calculation?

## 3 RESULTS

**FIGURE 1:**
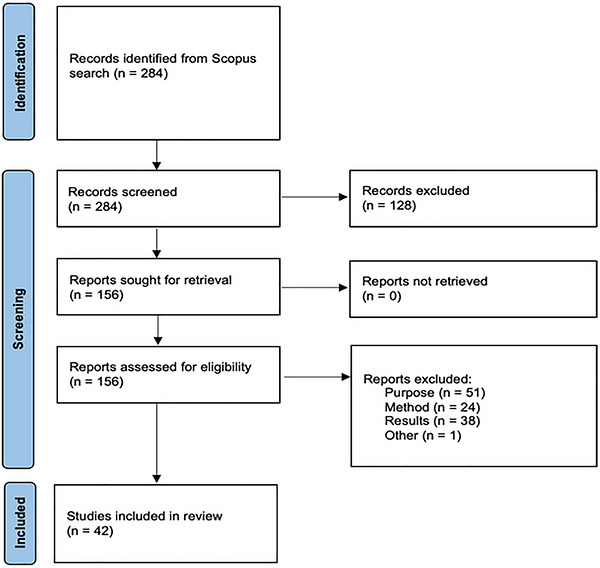
PRISMA flow diagram of the literature search and selection process for eligible studies on TG‐43, MBDCAs, and MC methods in brachytherapy.

The Scopus database was systematically searched, yielding 284 records in total. After screening titles and abstracts, 128 articles were excluded due to irrelevance. The remaining 156 full‐text articles were reviewed for eligibility, and 114 were excluded for reasons related to study purpose, methodology, or incomplete results. Ultimately, 42 studies met the inclusion criteria and were analyzed in this systematic review. The literature search and study selection process is summarized in Figure 1.

The full summary table of risk bias assessment can be found in Appendix D. Domains (3) and (4) consistently demonstrated low risk (+) across the literature, indicating that studies generally employed robust, clinically relevant metrics for validation and reported their findings and limitations transparently. More noticeable biases arise from domains (1) and (2). The only instance of high level of risk (+++) for all selected studies was observed in domain (2). This arose because the analytical RayStretch algorithm, which the study validated, intrinsically applied significant physical approximations by neglecting the effect of interseed attenuation and relying on an empirical scaling of the linear attenuation coefficient for modeling calcifications, rather than rigorous physics simulation.^28^ This imposed a fundamental limitation on the accuracy of the input model relative to physical reality. A recurring moderate risk (++) arose from a combination of limitations in CT‐based heterogeneity processing, geometric representation, and case representativeness across several studies. Issues related to density assignment and material modeling were reported in early MBDCA implementations. Terribilini et al.^29^ identified a flaw in a commercial MBDCA in which the scatter dose component was computed using default tissue densities rather than HU‐scaled physical densities derived from CT images. Similarly, Peppa et al.^30^, ^31^ reported that the treatment planning system (TPS) version used did not support automatic CT‐based density assignment, necessitating manual specification of uniform density and composition within regions of interest. Geometric resolution posed additional constraints: Desbiens et al.^32^ excluded interstitial needles and catheters from their simulations because these structures were smaller than the calculation grid resolution. Beyond modeling limitations, some studies relied on idealized or theoretical phantoms rather than patient‐specific anatomies, while others were constrained by small patient cohorts, such as the four anonymized cases analyzed by Ma et al.^33^ or the eight plans from five patients studied by Hyer et al.^34^. These constraints are best viewed as reflective of the developmental stage of MBDCAs and MC implementations during the study periods, rather than indicators of unreliable conclusions.

Overall, the identified sources of bias should be interpreted in the context of the technical and clinical complexity inherent to brachytherapy dose‐calculation research. Many of the observed limitations, such as simplified geometries, restricted patient cohorts, or approximations in material modeling, reflect practical constraints related to computational feasibility, historical TPS capabilities, and the evolving nature of heterogeneity‐aware algorithms, rather than methodological shortcomings unique to individual studies.

### 3.1 Dosimetric accuracy and clinical translation (RQ1)

Many studies ^23^, ^35^, ^36^, ^37^, ^38^, ^39^ agree that TG‐43 overestimates target coverage metrics (D_90_, V_100_) and misrepresents OAR doses in the presence of tissue heterogeneities or applicator materials. When heterogeneity modeling is introduced, target doses generally decrease by up to 5%, whereas small‐volume OAR metrics (D_0.1cc_, D_2cc_) vary significantly according to local geometry.

Comparisons between TG‐43 and water‐only MC implementations (TG‐43‐MC) show minimal differences, confirming that the observed discrepancies arise primarily from heterogeneity physics rather than transport formalism.^40^ Clinically, these findings imply that plans recalculated with MBDCAs or MC may appear less favorable under TG‐43‐based acceptance criteria despite being more physically accurate.

The representative quantitative differences between TG‐43 and MBDCAs/MC across major brachytherapy sites are summarized in Table 1.

**TABLE 1:** Representative dosimetric differences between TG‐43 and MBDCAs/MC.

Studies	Context	Site / Applicator	Reported change (MBDCA/MC vs. TG‐43)	Interpretation
^41^	Acuros BV vs. TG‐43 in LACC	Cervix HDR (T&O, titanium)	CTV_HR D_90_ ∼4% lower and OAR D_2cc_ 4%–6% lower for Acuros BV	Systematic reduction in target and OAR doses
^42^	ACE vs. TG‐43 for cervix HDR	Cervix HDR	HR‐CTV D_90_ difference ∼0.5%	Small but consistent directional change
^34^	TG‐186 vs. TG‐43 in HDR 192 Ir cervix	Cervix HDR	Up to ∼2% differences	Applicator material effects captured
^45^	CT‐based MC vs. TG‐43 for PIPB	Prostate LDR (I‐125)	D_90_ and Urethra D_0.1cc_ a few percent lower for MC	Inter‐seed attenuation reduces hot spots
^44^	MC vs. TG‐43 for Radiobiological analysis PIPB	Prostate LDR	D_90_ a few percents lower; BED/EQD2 follow trend	Lower isoeffective dose predicted
^31^	TG‐43 vs. MC for 192 Ir breast HDR	Breast HDR	PTV D_90_ 3%–5% lower; Skin D_2cc_ lower with MC	TG‐43 overestimates surface dose (due to lack of backscatter)
^49^	MC vs. TG‐43 dosimetry for oral cavity HDR	Head & Neck	V_200_ 19 ± 12% lower, NT D_2cc_ 5 ± 4% lower with MC	Pronounced air/bone interface effects

For cervical HDR treatments, several studies reported small but systematic reductions in high‐risk CTV coverage when switching from TG‐43 to Acuros BV or ACE. Specifically, in a large cohort comparison, Acuros BV yielded an average D_90_ reduction of approximately 4% and OAR D_2cc_ decreases of 4%–6% relative to TG‐43.^41^ Other studies using ACE or GBBS algorithms reported smaller changes (less than 1%–2%) but in the same direction.^34^, ^42^ Applicator modeling is particularly critical for dosimetric accuracy. Metallic tandems and Direction Modulation Brachytherapy (DMBT) catheters overestimated local dose errors up to 10% with TG‐43, which are corrected by MBDCAs recalculation.^34^ The most significant discrepancies occur with shielded applicators. Mikell et al. demonstrated that for shielded colpostats, MBDCAs (specifically GBBS) reveal a dose reduction to the rectum and bladder averaging 21.4% and 15.1% respectively, with maximum differences exceeding 25%.^43^ These findings underscore a critical clinical scenario where the TG‐43 water equivalent assumption fundamentally fails to account for the substantial attenuation of high‐*Z* shields, leading to significant overestimation of OAR doses. Although this review was not applicator‐specific, studies involving shielded applicators were included when they met the methodological eligibility criteria. These cases are particularly important because high‐*Z* shielding introduces attenuation effects not captured by TG‐43, although the resulting large deviations should be interpreted as scenario‐specific rather than representative of all HDR brachytherapy settings.

In permanent prostate implants, full‐physics MC simulations that incorporate inter‐seed attenuation and realistic tissue composition consistently report reductions of several percent in D_90_ and V_100_ compared with TG‐43, alongside lower urethral D_0.1cc_ values.^35^, ^44^, ^45^ These effects result from improved modeling of photon scatter, anisotropy, and attenuation that are not captured by the homogeneous water assumption of TG‐43.

For both HDR and seed‐based breast applications, TG‐43 tends to overestimate target coverage and near‐surface doses in the presence of lung or air interfaces. Recalculations with MC or MBDCAs consistently demonstrate PTV D_90_ reductions of approximately 3%–5% and skin D_2cc_ decreases of similar magnitude.^30^, ^31^, ^46^, ^47^, ^48^ These corrections are in better agreement with experimental film and TLD measurements, reinforcing the importance of heterogeneity‐aware dose calculation for accurate representation of patient anatomy.

Heterogeneity effects are most pronounced in head‐and‐neck sites, particularly within the oral cavity where air, bone, and dental materials coexist. A detailed MBDCAs study reported a 19 ± 12% reduction in V_200_ and a 5 ± 4% decrease in normal‐tissue D_2cc_ compared with TG‐43, while mucosal surface doses occasionally increased locally due to improved scatter modeling.^49^ These deviations exceed typical clinical tolerance levels and highlight the limitations of TG‐43 in high‐gradient, low‐density regions.

Differences in dose distributions propagate directly into derived radiobiological quantities such as the biologically effective dose (BED), equivalent dose in 2 Gy fractions (EQD2), tumor control probability (TCP), and normal‐tissue complication probability (NTCP). In cervix HDR studies, Acuros BV recalculations produced EQD2 reductions of approximately 4%–6% for the HR‐CTV and 3%–6% for OARs relative to TG‐43.^34^, ^41^, ^42^ In PIPB, MC‐based dosimetry resulted in BED and Equivalent Uniform Biologically Effective Dose (EUBED) reductions of about 5% and correspondingly lower TCP values at the same nominal prescription.^35^, ^44^

For breast applications, EQD2 decreased by 1%–5% with slight decreases in predicted skin NTCP near air or lung interfaces using MC.^31^, ^46^ In head‐and‐neck cases, using MC, lower EQD2 values were also observed for target volumes, with localized increases in mucosal surface dose consistent with the underlying heterogeneity physics.^49^ A summary of reported radiobiological differences between TG‐43 and MBDCAs/MC implementations across the reviewed studies is presented in Table 2.

**TABLE 2:** Reported radiobiological differences between TG‐43 and MBDCA/MC.

Studies	Context	Site / Model	Biological metric(s)	Reported difference	Interpretation
^41^	Acuros BV vs. TG‐43 (LACC)	Cervix HDR ± EBRT	EQD2 (target + OAR)	Target EQD2 4%–6% lower; OAR EQD2 3–6% lower with Acuros BV	Clinically meaningful EQD2 reduction
^44^	MC vs. TG‐43 for Radiobiological analysis PIPB	Prostate LDR	BED, EUBED, TCP	BED/EUBED ∼5% lower; TCP also slightly lower with MC.	Lower biological dose at fixed plan
^31^	TG‐43 vs. MC (192Ir breast HDR)	Breast HDR	EQD2, NTCP	EQD2 1%–5% lower; NTCP slightly lower with MC	Overestimated surface risk by TG‐43
^46^	103Pd breast LDR TG‐43 vs. MC comparison	Breast LDR	EUBED, NTCP	EUBED ∼6% lower; NTCP slightly lower with MC	Heterogeneity changes OAR risk balance
^49^	MC vs. TG‐43 oral cavity study	Head & neck HDR	EQD2 (derived from V_200_/D_2cc_)	EQD2 5%–10% lower with MC	Reflects interface‐driven redistribution

### 3.2 Validation and commissioning strategies (RQ2)

Many of the literature adopted the roadmap recommended by TG186^7^ in implementing MBDCAs in their clinics. For instance, ACE and Acuros frameworks demonstrated agreement within ± 3 % between Levels 1 (homogeneous water) and level 2 (applicator/bounded heterogeneities) scatter conditions.^29^, ^33^, ^34^ In the collapsed‐cone ACE validation, V_100_ deviations were less than 3% relative to MC, whereas TG‐43 deviations reached up to 10% in heterogeneous (air or lung) regions.^33^

MC engines such as egs_brachy, EGSnrc, and Geant4 served as the primary reference for both commissioning and validation of MBDCAs. Benchmark MC dose functions matched established literature values within ± 1%.^50^ These results confirm MC as the definitive reference standard for quantitative accuracy and for QA benchmarking during MBDCAs commissioning.^33^

Validation strategies for MBDCAs and MC methods commonly relied on experimental setups designed to reproduce clinically relevant heterogeneities, steep dose gradients, and shielding conditions that challenge the assumptions of the TG‐43 formalism.

Zwierzchowski et al.^51^ employed a 3D‐printed anthropomorphic head phantom composed of polylactic acid and plaster inserts to emulate various tissue heterogeneities in superficial head‐and‐neck HDR brachytherapy. The study incorporated lead shielding to further introduce high‐*Z* heterogeneities and sharp dose falloffs representative of clinical surface mold treatments. Dose distributions were measured using EBT3 radiochromic film. In this heterogeneous geometry, TG‐186–compliant MBDCA calculations agreed with film measurements within approximately 3%, whereas TG‐43 systematically overestimated dose by up to ∼10%, particularly in regions adjacent to air cavities and lead shielding.^51^

A cylindrical Perspex (PMMA) water phantom was employed by Ji et al.^52^ to validate dose calculations for an I‐125 esophageal brachytherapy stent using LiF:Mg,Cu,P TLD measurements at multiple radial depths. In this study, MC simulations consistently predicted the highest absorbed dose values, followed by the TG‐43–based TPS, with TLD measurements yielding slightly lower doses. The TPS implementation did not explicitly model material heterogeneities or applicator attenuation, whereas the MC calculations accounted for full photon transport physics. The reported absolute difference between MC and TLD measurements was less than 5 Gy across all evaluated depths, corresponding to approximately 7%–8% of the prescribed dose (∼60 Gy). This level of agreement is consistent with expected experimental uncertainties for low‐energy photon dosimetry.^52^

A summary of key validation setups, detectors, and their reported outcomes across the reviewed studies is presented in Table 3.

**TABLE 3:** Experimental validation methods.

Studies	Phantom / Geometry	Detector	Validation target	Key outcome
^51^	3D‐Printed head (PLA + Plaster)	EBT3 film	Superficial HDR with lead shield	MBDCA agreed with film measurements within ∼3%; TG‐43 overestimates ∼10%
^52^	Cylindrical perspex water	LiF:Mg, Cu, P TLD	I‐125 Esophageal stent	Doses calculated by MC > TPS > TLD; < 5 Gy absolute difference (7%–8%) between MC and TLD
^33^	Water & heterogeneous cases	Computational benchmark	ACE vs. TG‐43/MC	ACE (MBDCA) matched MC benchmark within < 3% difference for V100 DVH

### 3.2 Computational feasibility and workflow implications (RQ3)

Typical clinical plans require hours‐scale computation. Tai et al.^39^ reported that egs_brachy took approximately 4 h to simulate 10^8^ histories on an AMD Ryzen 9 5900X (12 cores). When implemented through eb_gui, preprocessing required ∼1 min and the main MC run ∼1 h for 3×10^9^ histories.^23^ In benchmark testing, RapidBrachyMC required 8416 s (∼2.3 h) for a representative breast plan, whereas the paired RapidBrachyTG‐43 calculation finished in 40–165 s on a 16‐thread Intel i7‐10700T CPU.^53^

For liver HDR applications, the ACE required 1‐h (Standard), 5‐h (High), and 6‐h (Custom) accuracy modes on a workstation equipped with 128 GB RAM and a Tesla K40c GPU. The reference MC run consumed ∼10^10^ histories.^37^ In broader multicase validations using Oncentra Brachy, dose‐recomputation times ranged from 10 to 70 min, depending on accuracy mode and dwell positions.^33^

In contrast, TG‐43 maintains second‐to‐minute‐level performance on standard clinical desktops. For the same benchmark geometry, RapidBrachyTG‐43 finished in 40–165 s versus ∼8416 s for MC.^53^

Emerging deep‐learning surrogates show orders‐of‐magnitude acceleration. RapidBrachyDL, a 3‐D CNN trained on MC dose maps, achieved a median 1.7 s per dwell prediction on CPU compared with 8.9 min per dwell for the 40‐CPU MC reference.^54^ Other DL models reported 1–6 s to produce prediction for parotid or prostate seed cases.^55^, ^56^ The representative computation times, number of simulated histories, and hardware configurations reported across the reviewed studies are summarized in Table 4.

**TABLE 4:** Representative computational performance data.

Studies	Engine/Method	Reported compute time	Histories	Hardware notes
^39^	MC (egs_brachy)	∼4 h	1×10^8^	AMD Ryzen 9 5900X (12 cores)
^23^	MC (egs_brachy via eb_gui)	1 min (prep); 1 h (dose)	3×10^9^	Development/validation workstation
^41^	MBDCA (AcurosBV)	∼43 s	—	BrachyVision clinical study
^53^	TG‐43 (RapidBrachyTG‐43)	40‐165 s	—	i7‐10700T (16 threads); paired MC 8416 s
^57^	MBDCA (ACE)	1 h standard; 5–6 h High/Custom	—	128 GB RAM; Tesla K40c GPU
^54^	DL surrogate (RapidBrachyDL)	∼1.7 s per dwell	—	MC ref ≈ 8.9 min per dwell (40 CPUs)
^33^	MBDCA (ACE validation)	10–70 min (case‐dependent)	5×10^7^‐5×10^9^ (MC ref)	ACE in Oncentra Brachy; MC on cluster

*Note*: Absolute computation times are heavily dependent on the specific CPU/GPU architectures of their respective eras. This table is not intended as a direct algorithmic benchmark, but rather to illustrate how technological advancements have reduced calculation times from hours to clinically acceptable minutes/seconds.

Implementing advanced MBDCA or MC engines in clinical practice requires not only computational resources but also changes in workflow and personnel infrastructure. First, Commissioning and validation efforts are substantial. For instance, multi‐institutional egs_brachy deployments required custom DICOM‐to‐MC pipelines, voxel‐wise material maps, and end‐to‐end comparisons against TPS outputs.^23^, ^40^ Retrospective replanning campaigns (e.g., 156 HDR fractions with AcurosBV) were performed to establish baseline behavior and clinical policy.^41^ To manage this complexity, integration and automation determine feasibility for day‐to‐day operation. Custom GUIs and scripts (e.g., eb_gui) allow rapid DICOM RT parsing and back‐transfer of DVH data.^23^ Furthermore, embedding ACE within Oncentra Brachy enables single‐environment computation with mode‐dependent timelines—standard accuracy matching clinical throughput and high/super‐high reserved for complex anatomies.^33^ In parallel, DL engines offer a complementary tool for rapid re‐dose and adaptive planning iterations.^54^, ^55^, ^56^ As automation improves, planning throughput improves substantially with standard‐mode MBDCA and DL surrogates, allowing interactive review and re‐optimization, whereas pure MC remains a bottleneck and is typically restricted to offline verification or research.^33^, ^41^, ^54^ Consequently, QA practices evolve accordingly. Initially, AcurosBV and ACE served as secondary verifiers for TG‐43 plans during transition phases; later, they became primary engines with MC or DL used for targeted secondary checks.^33^, ^41^, ^42^ Finally, training and reporting standards must also adapt. Reports cite the need for staff upskilling and updated SOPs, including consistent use of dose‐to‐medium versus dose‐to‐water metrics to avoid interpretation errors.^23^, ^40^, ^41^

The observed workflow impacts and representative supporting studies for each integration theme are summarized in Table 5.

**TABLE 5:** Workflow themes and representative evidence.

Studies	Workflow theme	Observed change in practice
^23^, ^40^, ^41^	Commissioning/Validation	Build DICOM pipelines; curate materials; end‐to‐end testing
^23^, ^33^, ^54^, ^56^	Integration/Automation	GUIs and scripts; ACE embedded in TPS; DL connectors
^33^, ^41^, ^54^	Planning Throughput	MBDCA standard inline; MC offline; DL for rapid what‐ifs
^33^, ^41^, ^42^	QA/Verification	MBDCA as primary; MC/DL as targeted secondary checks
^23^, ^40^, ^41^	Training/Policy	Staff upskilling; reporting conventions

### 3.3 Barriers and enablers for clinical adoption (RQ4)

Transitioning from the TG‐43 formalism to MBDCAs and MC methods remains constrained by a complex set of institutional, technical, and cultural barriers that collectively slow clinical translation. First and foremost, regulatory uncertainty and lack of unified standards remain a primary concern. Several studies highlight the absence of TG‐186‐consistent acceptance criteria and national audit pathways, leading to hesitation over non‐TG‐43 dose reporting.^32^, ^35^, ^39^, ^58^ Compounding this issue, resource constraints also limit implementation. Departments often face additional costs for workstation or cluster upgrades, TPS add‐on licenses, and the longer computation times required by advanced dose calculation engines.^41^, ^57^, ^59^, ^60^ Additionally, the commissioning and QA workload adds further pressure. End‐to‐end heterogeneous phantom tests and MC cross‐checks significantly expand acceptance and verification schedules, particularly during dual operation of TG‐43 and MBDCA workflows.^30^, ^54^, ^61^ Historically, this was exacerbated by a lack of independent benchmarking data; however, this specific barrier has been significantly mitigated by the AAPM TG‐372 report, which now provides standardized, pre‐vetted datasets for ^192^Ir HDR sources to facilitate robust commissioning.^62^ Beyond hardware and QA demands, persistent training and staffing gaps slow adoption. Limited expertise in transport physics, uncertainty analysis, and non‐TG‐43 planning underscores the need for structured competency frameworks and protected learning time.^33^, ^53^, ^55^ Simultaneously, workflow and IT integration issues—such as DICOM‐RT round‐tripping, HU‐to‐material mapping, and limited automation—remain practical bottlenecks.^14^, ^52^, ^63^, ^64^ These technical and educational hurdles are further exacerbated by concerns about deviating from TG‐43 conventions without established audit precedent.^31^, ^65^ Ultimately, cultural inertia perpetuates reliance on TG‐43. Clinicians are accustomed to historical TG‐43 dose metrics and inter‐center comparability, making them reluctant to recalibrate clinical constraints.^34^, ^51^, ^56^, ^66^

**FIGURE 2:**
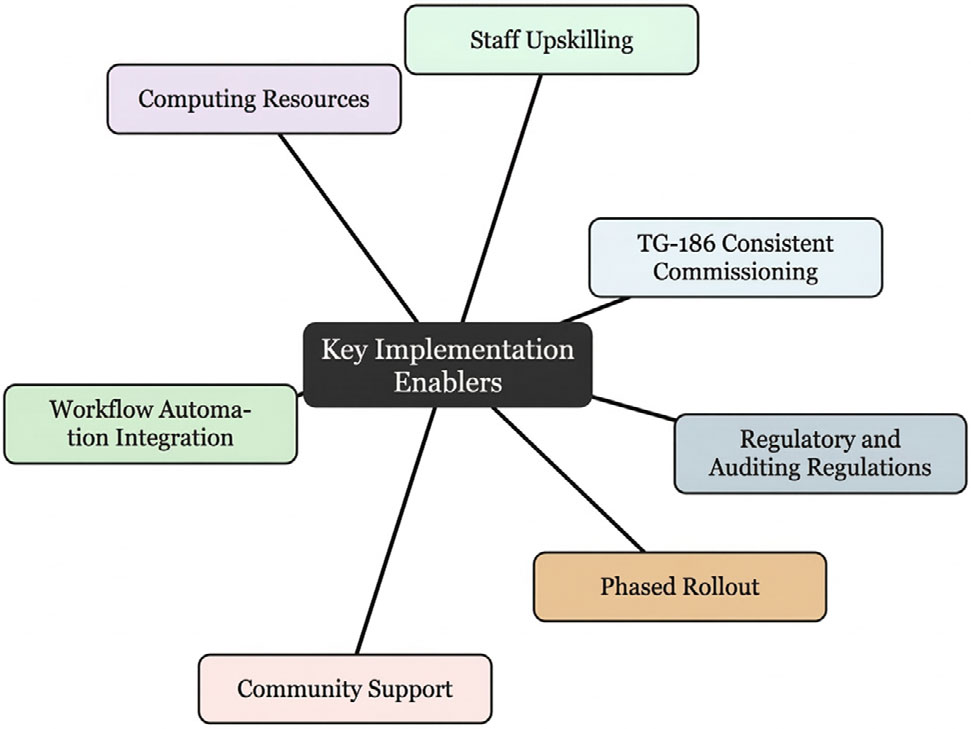
Key implementation enablers for MBDCAs/MC methods.

A primary enabler for successful use of MBDCAs or MC was the use of staged, TG‐186–consistent commissioning, which have been implemented via staged test cases and acceptance criteria: Groups operationalized TG‐186 using tiered validation (water‐only baselines followed by heterogeneous geometries), end‐to‐end phantom or benchmark comparisons, and site‐specific tolerance tables that were then reused for periodic QA and after software updates.^7^, ^43^, ^44^ This “test‐case first” approach functioned as a practical template for implementation and helped reduce inter‐clinic variability in interpreting non‐TG‐43 dose reporting. Furthermore, a safe rollout depended on structured staff upskilling (workshops, credentialing, and explicit guidance on dose‐to‐medium vs. dose‐to‐water interpretation), addressing the expertise gap repeatedly noted as a barrier.^38^, ^41^ In terms of technical infrastructure, clinical feasibility was enabled by appropriate computational resources (CPU/GPU capacity, parallelization strategies, and selecting “standard vs. high accuracy” modes when available), which allowed advanced calculations to fit within planning timelines.^28^, ^33^, ^42^, ^67^ Additionally, workflow‐level enablers include automation and data integration, leveraging DICOM‐RT QA scripts and templated reports to ensure reproducibility.^35^, ^46^, ^53^, ^68^ Beyond internal logistics, peer‐support and community normalization: multiple reports emphasize that adoption accelerates when clinics can rely on shared benchmarks, multi‐institutional experience, and community‐vetted implementation patterns (e.g., common phantom/test libraries and cross‐site comparisons), which increases confidence in non‐TG‐43 reporting and reduces the perceived medico‐legal risk of being an “outlier” center.^33^, ^59^, ^64^ Crucially, clinician trust was maintained through a phased rollout with dual reporting: Clinics maintained clinician trust by introducing MBDCAs/MC in parallel with TG‐43 for a defined transition period, using paired reporting to recalibrate expectations while preserving comparability with historical constraints.^29^, ^47^, ^51^ Collectively, these factors form a cohesive framework that facilitates clinical translation of advanced dose‐calculation algorithms. The main enabling factors are summarized in Figure 2.

### 3.4 Emerging innovations (RQ5)

Two principal innovation drivers were observed across the literature: The first major driver is GPU‐accelerated computation: Recent studies report significant runtime reduction by porting MC and grid‐based Boltzmann solvers to CUDA or OpenCL platforms. Such GPU‐based engines enable near‐real‐time recalculation suitable for adaptive planning and MC verification.^56^, ^67^ In parallel, the emergence of AI/deep‐learning dose surrogates: Neural‐network surrogates trained on MC‐quality datasets (e.g., RapidBrachyDL) reproduce MC‐level dose distributions in seconds, facilitating interactive optimization and online QA.^54^, ^63^

The integration of these computational advances is found to enable adaptive and personalized brachytherapy workflows. One primary benefit is the capability for adaptive/plan‐of‐the‐day recalculation, where **f**ast MBDCA/MC engines enable plan updates after anatomical or applicator changes, improving coverage consistency and OAR protection.^23^, ^30^, ^65^ Furthermore, the combination of dose accumulation and deformable registration yields heterogeneity‐aware DIR‐based accumulation yielding accurate cumulative DVHs, enhancing assessment of total dose from multiple insertions.^31^, ^57^, ^58^ These advancements also extend to personalized seed and needle guidance: Iterative MBDCA/MC feedback supports individualized seed geometry and needle trajectories, occasionally coupled with AI for auto‐planning.^38^, ^66^, ^67^ Finally, the synergy between AI‐assisted segmentation and optimization: Machine‐learning segmentation and constraint‐driven optimization significantly reduce planning time while maintaining dosimetric quality.^35^, ^41^, ^68^

Four priorities emerge from the reviewed literature. The first priority centers on real‐time adaptive recalculation: Achieving on‐table plan adaptation demands deterministic, low‐latency recalculation pipelines. GPU‐accelerated and AI‐assisted dose engines must be validated for timing, synchronization with imaging, and safety interlocks.^28^ Secondly, fostering AI‐MC synergy and robustness is crucial, as physics‐informed neural surrogates require training across diverse anatomies and applicator configurations. Multi‐institutional datasets are essential for generalization and uncertainty quantification.^53^, ^54^, ^66^ In this context, there are multi‐center benchmarks and outcomes linkages: Collaborative phantom libraries and shared benchmark repositories are needed to link dosimetric deviations (TG‐43 vs. MBDCA/MC) to clinical endpoints such as toxicity and tumor control.^44^, ^49^, ^68^ Finally, the field must prioritize radiobiological integration: Mapping dose‐to‐medium into radiobiological models (e.g., TCP/NTCP) will enable biologically adaptive optimization strategies unique to brachytherapy's dose‐rate dynamics.^32^, ^44^, ^46^ Future research should align computational acceleration with biological relevance—developing validated AI‐MC hybrids, standardized inter‐institutional benchmarks, and radiobiologically guided planning frameworks.

## 4 DISCUSSION

This systematic review synthesizes the existing evidence comparing the TG‐43 formalism with heterogeneity‐aware dose calculation approaches, including MBDCAs and MC methods, across multiple brachytherapy sites. The findings consistently demonstrate that while TG‐43 remains a practical and standardized framework for dose reporting, its simplifying assumptions can lead to systematic dose discrepancies in anatomically heterogeneous environments, as previously highlighted by both experimental and computational studies.^7^, ^8^, ^9^

Radiation dose deposition in brachytherapy is governed by photon interactions, primarily photoelectric absorption and Compton scatter, which create steep dose gradients around the source. Because clinical sources emit low‑ to intermediate‑energy photons, dose distributions are highly sensitive to local material composition, scatter conditions, and the presence or absence of charged particle equilibrium.^7^ The TG‑43 formalism simplifies transport by assuming an infinite water medium with full scatter, enabling fast calculation but neglecting real patient heterogeneity, finite patient size, and non‑water materials.^6^ In practice, tissues such as lung, bone, air cavities, and dental materials alter photon attenuation and scatter, producing localized dose differences relative to water assumptions. TG‑43 often overestimates dose near air interfaces due to missing backscatter, while applicator materials or shielding can cause TG‑43 to underestimate dose when real attenuation is greater than predicted. These effects vary by site and geometry, explaining why differences between TG‑43 and heterogeneity‑aware calculations do not follow a universal bias direction. Advanced algorithms such as deterministic model‑based dose‑calculation engines and MC methods explicitly model transport through patient‑specific materials and applicators. These methods capture inter‑seed attenuation in permanent implants, shielding effects in gynecologic applicators, and surface perturbations in breast or head‑and‑neck treatments. Because they account for the true transport environment, they generally produce dose distributions that differ from TG‑43 in both magnitude and direction across targets and OARs. These local physical differences propagate into DVH metrics and biological quantities such as EQD2, underscoring the importance of heterogeneity‑aware dose calculation in scenarios with significant material variation.

Across different anatomical sites, several investigations reported modest but directionally consistent reductions in target coverage when recalculating TG‐43‐based plans using MBDCAs or MC methods, typically on the order of 3%–6% for D90 metrics ^11^, ^12^, ^34^, ^41^, ^51^ These observations align with prior narrative reviews by Enger et al.^17^ and Yousif et al.,^18^ which emphasized the sensitivity of brachytherapy dose calculations to tissue heterogeneity and applicator composition. Importantly, localized dose perturbations adjacent to tissue–air interfaces, calcifications, or metallic applicators frequently exceeded 10%–20%, underscoring scenarios in which the TG‐43 water‐based assumption is most likely to break down. The magnitude of deviation near interfaces often exceeded the average global errors reported in DVH metrics, suggesting that commonly used summary parameters such as D_90_ or D_2cc_ may underrepresent clinically meaningful local differences. These local perturbations have been shown to propagate into radiobiological models, influencing derived EQD2, BED, TCP, and NTCP values. This is especially relevant for sites in which dose–response relationships are steep, such as cervical cancer and interstitial breast or oral cavity brachytherapy. As a result, reliance on TG‐43 in heterogeneity‐dense environments may lead to overestimation of tumor control probabilities or underestimation of toxicity risks, highlighting the importance of clinically integrating more accurate physics models wherever feasible.

In interpreting these findings, it is important to recognize that the dosimetric discrepancies observed between TG‐43 and heterogeneity‐aware algorithms are not merely technical artifacts but have direct implications for clinical decision‐making. Because TG‐43 systematically assumes full scattering conditions and ignores material composition, it tends to overestimate dose in regions where attenuation or reduced scatter significantly modifies the radiation field, as previously highlighted in TG‐186 and subsequent validation studies.^7^, ^16^ This becomes clinically consequential in anatomical sites with prominent air cavities (e.g., oral cavity), large bone structures (e.g., skull and mandible in head‐and‐neck implants), or high‐*Z* applicators (e.g., metallic tandems, surface molds with shielding). When dose is recalculated using MBDCAs or MC methods, treatment plans that appear to meet conventional TG‐43–based criteria may no longer satisfy planning objectives for target coverage, while OAR doses may shift upward or downward depending on local heterogeneity. These observations reinforce that transitioning to heterogeneity‐aware dose calculation requires not only dosimetric recalibration but also a thoughtful re‐evaluation of clinical constraints, reporting conventions, and acceptance criteria, consistent with prior recommendations.^7^, ^17^

Although dose discrepancies arising from tissue heterogeneity and applicator attenuation are the central focus of this review, they represent only one component of the overall uncertainty budget in brachytherapy. Other wellrecognized sources of variation include applicator reconstruction uncertainty, sourcepositioning deviations, catheter displacement during or between fractions, and organ motion or deformation, all of which can influence dose metrics at magnitudes comparable to or exceeding heterogeneity effects in some clinical scenarios. These geometric and positional uncertainties are particularly relevant in highgradient regions where small shifts can substantially modify D_2cc_, D_0.1cc_, or target coverage metrics. Placing heterogeneity corrections within this broader context highlights that MBDCA/MC approaches should complement, not replace, ongoing efforts to minimize applicator, source, and organposition uncertainties through improved imaging, verification, and qualityassurance procedures.

Because brachytherapy is a fastpaced and proceduredriven modality, clinical workflow directly influences the practicality of implementing modelbased and MC algorithms. Many centers continue to rely on forward planning, where physicians iteratively adjust dwell times and isodose distributions. In such settings, the feasibility of heterogeneityaware dose calculation depends on maintaining interactive planning speed, typically by using standardaccuracy MBDCA modes during realtime adjustments and reserving higheraccuracy or MC recalculations for final plan evaluation. The reported heterogeneitydriven differences in target and OAR metrics do not mandate universal prescription changes, but they do warrant site and geometryspecific review: clinicians may need to refine planning aims or adjust dwell patterns when significant deviations appear near interfaces or high‐*Z* materials. These workflowaware considerations support targeted, rather than wholesale, modifications to clinical practice as institutions transition beyond TG43.

Despite clear evidence supporting the superiority of MBDCAs/MC calculations, their implementation remains uneven across institutions. The reviewed studies point to several factors contributing to this translational gap. First, the absence of harmonized audit pathways and standardized acceptance tolerances for heterogeneity‐aware dosimetry remains a major barrier. Clinics accustomed to longstanding TG‐43 benchmarks may be reluctant to adopt new dose metrics without regulatory clarity. Second, the commissioning burden for MBDCAs and MC, requiring heterogeneous phantoms, cross‐platform benchmarking, DICOM integration workflows, and potentially large‐scale retrospective recalculations, can be substantial for departments with limited staffing or computational resources. It is because brachytherapy often requires same‑day plan approval, dose engines must support interactive iteration. In practice, deterministic MBDCAs embedded in TPSs enable inline, ‘standard‑mode’ recalculation during catheter optimization, with ‘high‑mode’ or MC reserved for approval or complex cases. Automation (templated HU‑to‑material assignment, scripted DVH export, and checklists) minimizes human latency. Centers using forward planning can phase in heterogeneity by dual reporting (TG‑43 and MBDCA) during transition, site‑specific tolerance tables, and mandatory heterogeneity checks at interfaces or near shields before plan approval. However, this hurdle is being systematically addressed by the recent release of the AAPM WGDCAB Report 372^62^ and the clinical test cases provided by Vasiliki et al.^69^. These resources offer standardized benchmarks and reference datasets for ^192^Ir HDR gynecologic brachytherapy, significantly reducing the local resource investment and technical expertise previously required for independent validation. Another important consideration is the challenge of interpreting dose‐to‐medium versus dose‐to‐water, especially in multi‐modality treatment contexts such as HDR brachytherapy combined with EBRT. While the physics community tends to endorse dose‐to‐medium for accuracy, many clinical protocols and historical dose–response data remain dose‐to‐water–based. This inconsistency complicates reporting and underscores the need for clear institutional policies and possibly consensus statements at the professional‐society level. Drawing a parallel to the transition from homogeneous to heterogeneous dose calculations in EBRT during the 1990s, the current shift in brachytherapy faces similar cultural inertia but encounters unique physical challenges. Unlike the relatively rapid adoption of heterogeneity corrections in EBRT, brachytherapy has faced a much slower transition despite TG‐186 being published over a decade ago. This disparity is driven by the extreme dose gradients inherent in brachytherapy, the high sensitivity to the positioning and modeling of high‐*Z* shielded applicators, and the historical lack of standardized commissioning benchmarks. Similarly, clinician acceptance requires adequate training to build confidence in the new algorithms, especially when previously accepted plans appear “worse” following MBDCAs or MC recalculation despite being more physically accurate.

## 5 FUTURE DIRECTIONS

Looking ahead, several developments are poised to advance the clinical integration of heterogeneity‐aware brachytherapy dose calculation. First, there is a clear need for standardized, multi‐center commissioning and validation frameworks. Although TG‐186 provides high‐level guidance, practical implementation varies widely across institutions. The development of shared benchmark datasets, heterogeneous phantom libraries, and community‐validated acceptance criteria would help reduce inter‐institutional variability and increase confidence in dose reporting beyond TG‐43. In this context, a formal survey led by professional societies such as the AAPM or American Brachytherapy Society (ABS) would be a valuable next step to accurately tally clinical adoption rates and identify the specific institutional barriers that persist across the community.

Future progress also depends on deeper integration of radiobiological modeling with accurate dose calculation. As MBDCAs and MC methods more reliably estimate dose in heterogeneous tissues, radiobiological frameworks must evolve to incorporate dose‐to‐medium information and account for spatially varying heterogeneity effects. Prospective studies linking recalculated doses to clinical outcomes, such as toxicity, tumor control, and local recurrence, are essential for translating dosimetric improvements into evidence‐based updates to planning constraints. Efforts to unify dose‐to‐water and dose‐to‐medium conventions within both clinical and research contexts will, likewise, be critical to ensure consistent interpretation across modalities.

At the technological level, rapid computation through GPU‐accelerated engines and deep‐learning surrogate models represents an important frontier. These tools have already demonstrated dramatic reductions in MC runtimes and show potential for real‐time or near–real‐time planning and verification. As hybrid AI–MC workflows mature, they may reduce the barrier of implementation. Continued evaluation of model generalizability, uncertainty quantification, and safety validation will be necessary to support their clinical deployment.

## 6 CONCLUSION

This systematic review demonstrates that the TG‐43 formalism, systematically misrepresents dose in heterogeneous brachytherapy scenarios. In contrast, MBDCAs and MC methods provide more accurate and clinically relevant dose estimates, with experimental and computational evidence consistently showing agreement within approximately 3% of reference data. These improvements also yield more reliable biological metrics, reinforcing the clinical value of heterogeneity‐aware planning.

Successful adoption of MBDCAs and MC methods depends on structured commissioning aligned with TG‐186, appropriate computational resources, and clear institutional workflows for QA, reporting, and staff training. Although modern hardware and emerging AI‐based accelerators have reduced computation times to clinically feasible levels, the absence of standardized audit pathways, persistent workflow complexity, and variability in clinical expertise continue to limit widespread integration.

Future progress will require harmonized validation frameworks, clearer regulatory guidance, and stronger evidence linking improved dose accuracy to clinical outcomes.

## AUTHOR CONTRIBUTIONS

**David A. Bradley**: Methodological guidance; quality assurance framework development; and critical revision of the manuscript for intellectual content. **James C. L. Chow**: Overall supervision; technical validation; interpretation of results; and final review and approval of the manuscript. **Thanh‐Tai Duong**: Conceptualization; study design; data curation; data extraction; data analysis; manuscript drafting; project supervision; and final approval of the manuscript. **Shada Wadi‐Ramahi**: Clinical validation; interpretation of oncologic relevance; and critical review of the manuscript for clinical accuracy and clarity. **Dat Tran**: Literature review; data extraction; data synthesis; preparation of figures and tables; and manuscript drafting.

## CONFLICT OF INTEREST STATEMENT

The authors declare no conflicts of interest.

## ETHICAL APPROVAL

Not applicable. This systematic review used previously published studies and did not involve new human participants, animal experiments, or patient‐identifiable data.

## DISCLOSURES

All authors declare that they have nothing to disclose.

## Supporting information




**Supporting information**: acm270627‐supp‐0001‐appendixA‐D.docx

## Data Availability

All data supporting the findings of this systematic review are available within the article and its supplementary materials.
