# Supplementary material for: Beyond TG‑43: A PRISMA‐based systematic review on model‐based dose‐calculation algorithms in brachytherapy
Source: J Appl Clin Med Phys. 2026 May 20;27(5):e70627. doi: 10.1002/acm2.70627 (PMC13240580; doi:10.1002/acm2.70627)
Supplement: Supplementary file 1 — Supporting information: acm270627‐supp‐0001‐appendixA‐D.docx [file ACM2-27-e70627-s001.docx]

Review Article

**Beyond TG‑43: A PRISMA-Based** **Systematic Review on Model-Based Dose-Calculation Algorithms in Brachytherapy**

**12. Supplementary Material**

**Appendix A: List of Abbreviations**

| **Abbreviation** | **Definition** |
| --- | --- |
| **AAPM** | American Association of Physicists in Medicine |
| **ACE** | Advanced Collapsed-Cone Engine (Elekta) |
| **AI** | Artificial Intelligence |
| **APBI** | Accelerated Partial Breast Irradiation |
| **ART** | Adaptive Radiotherapy |
| **BED** | Biologically Effective Dose |
| **CCS** | Collapsed Cone Superposition |
| **CNN** | Convolutional Neural Network |
| **CPU** | Central Processing Unit |
| **CTV** | Clinical Target Volume |
| **CT** | Computed Tomography |
| **CUDA** | Compute Unified Device Architecture (NVIDIA GPU platform) |
| **D2cc** | Dose to the most exposed 2 cm³ of an organ at risk |
| **D90** | Dose covering 90 percent of the clinical target volume |
| **DL** | Deep Learning |
| **DIR** | Deformable Image Registration |
| **DMBT** | Direction Modulation Brachytherapy |
| **Dm,m** | Dose-to-Medium-in-Medium |
| **Dw,w** | Dose-to-Water-in-Water |
| **DVH** | Dose–Volume Histogram |
| **DVH-QA** | Dose–Volume Histogram-based Quality Assurance analysis |
| **EBRT** | External Beam Radiotherapy |
| **egs_brachy** | Open-source Monte Carlo code for brachytherapy dose calculation (based on EGSnrc) |
| **EGSnrc** | Electron Gamma Shower National Research Council Monte Carlo system |
| **EQD2** | Equivalent Dose in 2 Gy Fractions |
| **EUBED** | Equivalent Uniform Biologically Effective Dose |
| **GBBS** | Grid-Based Boltzmann Solver |
| **Geant4** | General-purpose Monte Carlo toolkit for particle transport |
| **GPU** | Graphics Processing Unit |
| **HDR** | High-Dose-Rate Brachytherapy |
| **HU** | Hounsfield Unit |
| **HU-ρ** | Hounsfield Unit-to-Density Calibration |
| **IOP** | Intra-Operative Planning |
| **LACC** | Locally Advanced Cervical Cancer |
| **LDR** | Low-Dose-Rate Brachytherapy |
| **MC** | Monte Carlo |
| **MC-DL** | Hybrid Monte Carlo–Deep Learning dose engine |
| **MC-GPU** | Monte Carlo implementation on GPU architecture |
| **MBDCAs** | Model-Based Dose-Calculation Algorithms |
| **MCNP** | Monte Carlo N-Particle Code |
| **ML** | Machine Learning |
| **MOSFET** | Metal–Oxide–Semiconductor Field-Effect Transistor |
| **NTCP** | Normal-Tissue Complication Probability |
| **OAR** | Organ at Risk |
| **PIPB** | Permanent Implant Prostate Brachytherapy |
| **PLA** | Polylactic Acid |
| **PMMA** | Polymethyl Methacrylate |
| **PRISMA** | Preferred Reporting Items for Systematic Reviews and Meta-Analyses |
| **QA** | Quality Assurance |
| **QA-DL** | Quality Assurance using Deep Learning surrogates |
| **RAID** | Redundant Array of Independent Disks (computing architecture) |
| **RHC** | Right-Handed Coordinate (geometry context) |
| **RT** | Radiotherapy |
| **TCP** | Tumor Control Probability |
| **TG-43** | AAPM Task Group No. 43 (Brachytherapy Dose-Calculation Formalism) |
| **TG-186** | AAPM Task Group No. 186 (Model-Based Dose-Calculation Methods) |
| **TG-372** | AAPM Task Group No. **372 (Commissioning of model-based dose calculation algorithms in brachytherapy (2023)** |
| **TLD** | Thermoluminescent Dosimeter |
| **TPS** | Treatment Planning System |
| **V100** | Volume receiving 100 percent of the prescription dose |
| **V200** | Volume receiving 200 percent of the prescription dose |
| **VoxMC** | Voxel-based Monte Carlo dose calculation platform |

**Appendix B: Summary of Contribution to Research Questions**

| **Study** | **Authors** | **RQ1** | **RQ2** | **RQ3** | **RQ4** | **RQ5** |
| --- | --- | --- | --- | --- | --- | --- |
| [46] | Afsharpour, et al. | ✓ | ✓ | ✓ | ✓ | ✓ |
| [3] | Akhavanallaf, et al. | ✓ | ✓ | ✓ | ✓ | ✓ |
| [5] | Bi, et al. | ✓ |  | ✓ | ✓ | ✓ |
| [6] | Chatzipapas, et al. | ✓ | ✓ | ✓ | ✓ | ✓ |
| [7] | Deering, et al. | ✓ |  | ✓ | ✓ | ✓ |
| [8] | Desbiens, et al. | ✓ | ✓ | ✓ | ✓ | ✓ |
| [10] | Duque, et al. | ✓ | ✓ | ✓ | ✓ | ✓ |
| [11] | Eason, et al. | ✓ | ✓ | ✓ | ✓ | ✓ |
| [13] | Famulari, et al. | ✓ | ✓ | ✓ | ✓ | ✓ |
| [14] | Fionda, et al. | ✓ |  | ✓ | ✓ | ✓ |
| [15] | Fotina, et al. | ✓ |  | ✓ | ✓ | ✓ |
| [16] | Fuse, et al. | ✓ | ✓ | ✓ | ✓ | ✓ |
| [17] | Haidari, et al. | ✓ |  | ✓ | ✓ | ✓ |
| [18] | Howie, et al. | ✓ | ✓ | ✓ | ✓ | ✓ |
| [19] | Hueso-González, et al. | ✓ | ✓ | ✓ | ✓ | ✓ |
| [20] | Hyer, et al. | ✓ | ✓ | ✓ | ✓ | ✓ |
| [21] | Ji, et al. | ✓ | ✓ | ✓ | ✓ | ✓ |
| [22] | Kalinowski, et al. | ✓ | ✓ | ✓ | ✓ | ✓ |
| [24] | Ma, et al. | ✓ | ✓ | ✓ | ✓ | ✓ |
| [25] | Mao, et al. | ✓ | ✓ | ✓ | ✓ | ✓ |
| [26] | Mazur, et al. | ✓ | ✓ | ✓ | ✓ | ✓ |
| [27] | Meftahi, et al. | ✓ | ✓ | ✓ | ✓ | ✓ |
| [28] | Miksys, et al. | ✓ | ✓ | ✓ | ✓ | ✓ |
| [29] | Miksys, et al. | ✓ | ✓ | ✓ | ✓ | ✓ |
| [30] | Mishra, et al. | ✓ | ✓ | ✓ | ✓ | ✓ |
| [31] | Moghadam, et al. | ✓ | ✓ | ✓ | ✓ | ✓ |
| [32] | Moghadam, et al. | ✓ | ✓ | ✓ | ✓ | ✓ |
| [35] | Peppa, et al. | ✓ | ✓ | ✓ | ✓ | ✓ |
| [36] | Peppa, et al. | ✓ |  | ✓ | ✓ | ✓ |
| [37] | Radcliffe, et al. | ✓ |  | ✓ | ✓ | ✓ |
| [39] | Schaller, et al. | ✓ |  | ✓ | ✓ | ✓ |
| [40] | Sinnatamby, et al. | ✓ | ✓ | ✓ | ✓ | ✓ |
| [41] | Sinnatamby, et al. | ✓ | ✓ | ✓ | ✓ | ✓ |
| [42] | Srivastava, et al. | ✓ |  | ✓ | ✓ | ✓ |
| [43] | Tai, et al. | ✓ | ✓ | ✓ | ✓ | ✓ |
| [44] | Terribilini, et al. | ✓ | ✓ | ✓ | ✓ | ✓ |
| [46] | Tian, et al. | ✓ | ✓ | ✓ | ✓ | ✓ |
| [47] | Xiao, et al. | ✓ | ✓ | ✓ | ✓ | ✓ |
| [48] | Xiong, et al. | ✓ | ✓ | ✓ | ✓ | ✓ |
| [50] | Zourari, et al. | ✓ |  | ✓ | ✓ | ✓ |
| [51] | Zwierzchowski, et al. | ✓ | ✓ | ✓ | ✓ | ✓ |

**Appendix C: Full Search String**

**TITLE-ABS-KEY** (

(brachyther* OR "interstitial brachytherapy" OR "intracavitary brachytherapy" OR "HDR brachytherapy" OR "LDR brachytherapy" OR "cervical brachytherapy" OR "prostate brachytherapy" OR "breast brachytherapy" OR "head and neck brachytherapy")

**AND**

("TG-43" OR TG43 OR "Task Group 43" OR "AAPM TG-43" OR "TG-43 formalism" OR "TG-43 protocol" OR "TG-186" OR TG186 OR "Task Group 186" OR "AAPM TG-186" OR "model-based dose calculat*" OR MBDCA OR "model-based algorithm*" OR Acuros OR "Acuros BV" OR "ACE algorithm" OR GBBS OR "grid-based Boltzmann solver" OR "Monte Carlo" OR "MC simulation" OR "MC dose calculat*" OR "MC code" OR EGSnrc OR egs_brachy OR DOSXYZnrc OR Geant4 OR GATE OR TOPAS OR MCNP OR PENELOPE OR FLUKA)

**AND**

(D90 OR V100 OR V150 OR V200 OR D2cc OR D0.1cc OR "conformity index" OR "conformity number" OR "homogeneity index" OR HI OR DVH OR "dose volume histogram" OR BED OR EQD2 OR TCP OR "tumor control probability" OR NTCP OR "normal tissue complication probability" OR "clinical outcome*" OR toxicity OR complication* OR "local control" OR survival OR OS OR PFS OR "treatment adaptation" OR "adaptive planning" OR "quality assurance" OR QA OR verification OR validation OR benchmarking OR "code benchmarking" OR "phantom stud*" OR "film dosimetry" OR "radiochromic film" OR "Gafchromic film" OR TLD OR OSLD OR "in vivo dosimetry" OR audit OR "end-to-end test")

**AND**

("computational feasibility" OR "calculation time" OR runtime OR "processing time" OR efficiency OR "clinical workflow" OR "workflow integration" OR "clinical implementation" OR "implementation feasibility" OR "treatment planning system" OR TPS OR Oncentra OR Eclipse OR BrachyVision OR "GPU acceleration" OR GPU OR "real-time" OR "fast Monte Carlo" OR "accelerated computation" OR "artificial intelligence" OR AI OR "machine learning" OR ML OR "deep learning" OR "neural network*" OR CNN OR "dose prediction" OR "dose estimation" OR "automated planning" OR "auto-contouring" OR "adaptive brachytherapy" OR "adaptive workflow" OR "online adaptive" OR "real-time adaptation" OR IGABT OR "personalized brachytherapy" OR "barriers to adoption" OR "clinical adoption" OR "implementation barrier*" OR "regulatory challenge*" OR "guideline adoption" OR "training challenge*" OR "resource limitation*" OR "clinical acceptance" OR "institutional adoption" OR "clinical uptake"))

**AND** (PUBYEAR > 2011) **AND** (LIMIT-TO(LANGUAGE, "English")) **AND** (LIMIT-TO (SRCTYPE, "j"))

**Appendix D: Risk of Bias Assessment**

| **Study** | **Authors** | **Geometry and Case Representativeness** | **Input Modeling and Material Assignment** | **Outcome Measurement and Validation** | **Selective Outcome Reporting** |
| --- | --- | --- | --- | --- | --- |
| [46] | Afsharpour, et al. | + | + | + | + |
| [3] | Akhavanallaf, et al. | + | + | + | + |
| [5] | Bi, et al. | + | + | + | + |
| [6] | Chatzipapas, et al. | ++ | + | + | + |
| [7] | Deering, et al. | + | + | + | + |
| [8] | Desbiens, et al. | + | ++ | + | + |
| [10] | Duque, et al. | + | + | + | + |
| [11] | Eason, et al. | ++ | ++ | + | + |
| [13] | Famulari, et al. | ++ | ++ | + | + |
| [14] | Fionda, et al. | ++ | + | + | + |
| [15] | Fotina, et al. | ++ | + | + | + |
| [16] | Fuse, et al. | + | + | + | + |
| [17] | Haidari, et al. | ++ | + | + | + |
| [18] | Howie, et al. | + | + | + | + |
| [19] | Hueso-González, et al. | + | ++ | + | + |
| [20] | Hyer, et al. | ++ | +++ | + | + |
| [21] | Ji, et al. | ++ | + | + | + |
| [22] | Kalinowski, et al. | + | ++ | + | + |
| [24] | Ma, et al. | ++ | + | + | + |
| [25] | Mao, et al. | ++ | + | + | + |
| [26] | Mazur, et al. | + | ++ | + | + |
| [27] | Meftahi, et al. | + | ++ | + | + |
| [28] | Miksys, et al. | ++ | + | + | + |
| [29] | Miksys, et al. | + | + | + | + |
| [30] | Mishra, et al. | + | + | + | + |
| [31] | Moghadam, et al. | ++ | + | + | + |
| [32] | Moghadam, et al. | ++ | + | + | + |
| [35] | Peppa, et al. | + | ++ | + | + |
| [36] | Peppa, et al. | + | + | + | + |
| [37] | Radcliffe, et al. | + | ++ | + | + |
| [39] | Schaller, et al. | + | + | + | + |
| [40] | Sinnatamby, et al. | + | + | + | + |
| [41] | Sinnatamby, et al. | + | + | + | + |
| [42] | Srivastava, et al. | + | + | + | + |
| [43] | Tai, et al. | ++ | + | + | + |
| [44] | Terribilini, et al. | + | ++ | + | + |
| [46] | Tian, et al. | ++ | ++ | + | + |
| [47] | Xiao, et al. | ++ | + | + | + |
| [48] | Xiong, et al. | + | + | + | + |
| [50] | Zourari, et al. | + | ++ | + | + |
| [51] | Zwierzchowski, et al. | + | + | + | + |
